# Editorial: *Clostridium difficile* infection in the Asia-Pacific region

**DOI:** 10.3389/fcimb.2022.983563

**Published:** 2022-07-26

**Authors:** Dazhi Jin, Yi-Wei Tang, Thomas V. Riley

**Affiliations:** ^1^ School of Laboratory Medicine, Hangzhou Medical College, Hangzhou, China; ^2^ Key Laboratory of Biomarkers and In Vitro Diagnosis Translation of Zhejiang province, Hangzhou, China; ^3^ Institute of Clinical Microbiology, Hangzhou Medical College, Hangzhou, China; ^4^ Division of Medical Affairs, Danaher Diagnostic Platform/Cepheid, Shanghai, China; ^5^ School of Biomedical Sciences, The University of Western Australia, Perth, WA, Australia; ^6^ Department of Microbiology, PathWest Laboratory Medicine (WA), Perth, WA, Australia

**Keywords:** *Clostridioides difficile*, molecular epidemiology, Asia-Pacific region, genotype, clinical features

*Clostridioides* (*Clostridium*) *difficile* has been recognized as a leading pathogen responsible for antimicrobial-associated diarrhea worldwide. *C. difficile* infection (CDI) results in a variety of clinical manifestations, ranging from asymptomatic carriage to severe life-threatening sepsis, toxic megacolon and pseudomembranous colitis. The molecular epidemiology of CDI has been well documented in North America, Europe and Australia, and *C. difficile* isolated from these regions has been studied in detail with different molecular types of *C. difficile* circulating. There were approximately 250,000 CDI cases, 13,000 deaths and medical costs of US$1 billion in the United States in 2017 (http://dx.doi.org/10.15620/cdc:82532). However, CDI has been largely under-studied in the Asia-Pacific region and the few studies that have been conducted suggest a different population of *C. difficile* in this region and unique characteristics of CDI. Thus, we organized a Research Topic in “Frontiers in Cellular and Infection Microbiology” in 2019 which included 8 peer-reviewed articles providing a historical overview of CDI in general, as well as summarizing genomic, epidemiological and clinical features of CDI in the Asia-Pacific region. Due to the growing interest in *C. difficile* in the region, in October, 2021, we were invited to arrange another Research Topic to highlight more novel findings. Of great interest were papers about country-specific epidemiology and papers featuring clinical pictures from different countries in the Asia-Pacific region.

So far, four articles have been successfully published in this Research Topic including two systematic reviews and two research papers contributed by the 27 authors. The Research Topic has been viewed over 3,000 times so far, each of the four articles has been accessed over 1,000 times. There is a systematic review and meta-analysis, derived from 79 studies conducted between 1981 and 2019, authored by Rodriguez-Palacios et al., which revealed sources of heterogeneity, high-risk foods and an unexpected higher prevalence of *C. difficile* in tropical areas. Luo et al. reviewed CDI prevalence in hematopoietic stem cell transplant (HSCT) patients in 43 studies from 2014 to 2021. The above two reviews comprehensively summarized the distribution of *C. difficile* in the human diet and updated important features about CDI among HSCT recipients, a significant contribution to CDI epidemiology worldwide. The remaining two articles described the molecular epidemiology of CDI in Eastern China, including risk factors for the dominant multi-locus sequence typing (ST) 81 (Yang et al.), and an atypical septicemia caused by *C. difficile* contributed by Wang et al. A *C. difficile* ST54 strain that was toxin A negative and toxin B positive and genetically related to ST37 was reported from China because of significant antimicrobial resistance and sporulation capacity. This was the first report of a monomicrobial bloodstream infection caused by *C. difficile* ST54 in China and provided important information for clinicians.

*C. difficile* is now being recognized as an important cause of healthcare-associated infection in the Asia-Pacific region possibly because of rapid economic growth, the aging population, and continuing inappropriate use of antimicrobials. However, *C. difficile* is often still neglected in the diagnosis and treatment of patients with diarrhea in the region, and CDI cases are often missed since clinicians have poor awareness with inappropriate testing being used. To date, relatively limited data have still been available on the epidemiology, genome evolution, prevalence, and impact of CDI in the Asia-Pacific region. Current literature shows that the molecular characteristics of *C. difficile* in the Asia-Pacific region are different from those in North America, Europe, and Australia. Hypervirulent *C. difficile* ribotype 027/ST1 and other binary toxin-producing *C. difficile* strains have rarely been described in this region. Genome-based phylogenetic clade 4, including *C. difficile* ST37, ST81, ST369, and others, was the predominant genotype. Despite the CDI prevalence in this region being similar to that in other continents, there have been few reports of severe outcomes of CDI (https://doi.org/10.1128/JCM.01217-20). However, some recent studies have described severe CDI cases significantly associated with the ST37 genotype (https://doi.org/10.1128/JCM.01898-16), and the sepsis case induced by ST54 reported in this Research Topic suggests that better surveillance for severe CDI in the Asia-Pacific region is required. In addition, it has been reported that age as a risk factor for CDI was much lower in China (less than 60 years old) than that seen in developed countries (https://doi.org/10.1128/JCM.01898-16). Given the population in the Asia-Pacific region comprises approximately 60% of the world’s population, and it is aging, the possibility of a *C. difficile*-related health crisis exists.

The four articles published so far on this Research Topic present meaningful CDI data, and we hope that more scientists and clinicians will focus on CDI studies from the Asia-Pacific region. There is still a variety of questions on CDI in the Asia-Pacific region that require answers, and these should be addressed in a systematic manner. What are the main transmission pathways for CDI? What are the genomic characteristics of Asian *C. difficile* and how has the genome evolved? What is known about *C. difficile* in animals in the Asia-Pacific region and, most importantly, the environment? Are there any correlations between contaminated food and CDI? Are traditional dietary habits in the region risk factors for CDI or *C. difficile* colonization, or are they possibly protective? Consequently, we are going to continue promoting this Research Topic, to attract more contributors, and to focus on the above questions in order to better understand the epidemiology, prevalence and medical burden of CDI in the Asia-Pacific region.

## Author contributions

DJ drafted the manuscript. TVR and Y-WT revised and edited the Editorial. All authors approved the final version of the manuscript.

## Conflict of interest

Author Y-WT is employed by Danaher Diagnostic Platform/Cepheid.

The remaining authors declare that the research was conducted in the absence of any commercial or financial relationships that could be construed as a potential conflict of interest.

## Publisher’s note

All claims expressed in this article are solely those of the authors and do not necessarily represent those of their affiliated organizations, or those of the publisher, the editors and the reviewers. Any product that may be evaluated in this article, or claim that may be made by its manufacturer, is not guaranteed or endorsed by the publisher.

